# Disease Burden and Treatment Patterns Associated With Eosinophilic Esophagitis in the United States: A Retrospective Claims Study

**DOI:** 10.1097/MCG.0000000000001491

**Published:** 2021-01-12

**Authors:** Mei Lu, Bridgett Goodwin, Montserrat Vera-Llonch, James Williams

**Affiliations:** * Shire, A Takeda Company, Lexington; † Shire, A Takeda Company, Cambridge, MA

**Keywords:** eosinophilic esophagitis, disease burden, treatment patterns

## Abstract

This US-based, retrospective claims study aimed to investigate disease burden and treatment patterns in patients with eosinophilic esophagitis (EoE), and to compare health care resource use (HCRU) in patients with EoE and matched controls without EoE.

**Materials and Methods:**

Patients with a diagnosis of EoE and ≥12 months of prediagnosis data were identified from the Truven Health MarketScan Research databases (January 2008 to September 2016) and followed up from the diagnosis date until termination of eligibility for a health plan. Patient clinical characteristics and HCRU were recorded in the 12 months before diagnosis; HCRU and treatment patterns were recorded during follow-up. HCRU in patients with EoE and matched controls was compared during the 12-month postdiagnosis period.

**Results:**

Among the 23,003 patients with EoE (mean age: 34.3 y; 64.8% male), gastroesophageal reflux disease was the most common prediagnosis condition (34.6%). After diagnosis, the most common off-label, first-line treatments were proton pump inhibitor monotherapy (52.8%) and topical corticosteroid monotherapy (21.5%). Overall, 3336 patients (14.5%) received at least 3 lines of off-label pharmacotherapy. Outpatient visits (recorded in 99.9% of patients on and postdiagnosis) were most frequently to gastroenterologists/pediatric gastroenterologists (49.5% prediagnosis, 72.6% on and postdiagnosis). Inpatient admissions and outpatient and emergency room visits were more likely in patients with EoE than in matched controls (*P*<0.0001).

**Conclusions:**

Patients with EoE in the USA experience a high disease burden both before and after diagnosis, which requires significant HCRU. Our findings highlight the unmet need for adequate control of EoE-related symptoms.

## Abstract 2

Supplemental Digital Content is available in the text.

Eosinophilic esophagitis (EoE) is a chronic immune-mediated disease characterized by esophageal inflammation^1^ leading to dysphagia, esophageal stricture and food impaction in adults, and reflux-like symptoms and feeding problems in children.^2^,^3^ The estimated prevalence of EoE in the USA is 0.4 to 0.9 cases per 1000 people, and ∼10 to 13 new cases per 100,000 people are diagnosed each year.^4^ Studies of the epidemiology of EoE in Western countries have reported an increase in prevalence in recent decades, primarily owing to increasing incidence, growing awareness of the condition, and more frequent use of diagnostic endoscopy in clinical practice.^4^–^7^

Up to 80% of patients with EoE have a history of atopic disease, such as asthma, allergic rhinitis, or food allergy.^3^,^8^ Over 90% of adults with EoE have intermittent dysphagia for solid food and ∼60% experience food impaction.^3^ In addition to these symptoms, children with EoE may experience vomiting, and chest and abdominal pain.^9^,^10^ There are currently no licensed therapies for EoE in the USA. Clinical guidelines from the American Gastroenterological Association and the Joint Task Force (AGA/JTF) on Allergy-Immunology Practice Parameters recommend treatment with proton pump inhibitors (PPIs) (conditional recommendation, very low quality of evidence) or swallowed topical corticosteroids (TCS) (strong recommendation, moderate quality of evidence) and the elimination of food allergens as first-line therapies for EoE.^11^ Neither PPIs nor TCS are specifically indicated for the treatment of EoE in the USA, thus are used off-label, but have demonstrated clinical benefits in patients with EoE.^11^ In adult patients with dysphagia from a stricture associated with EoE, AGA/JTF recommends esophageal dilation (conditional recommendation, very low quality of evidence).^11^

Data from clinical trials^12^,^13^ and retrospective cohort studies^14^,^15^ have demonstrated that in the absence of treatment EoE does not spontaneously resolve; endoscopic signs and esophageal eosinophilia recur in almost all patients when treatment is discontinued.^4^,^16^ The ongoing requirement for dietary restrictions and maintenance treatment, and fear of food impaction, can disrupt daily and social activities and impair health-related quality of life, particularly in patients who experience severe symptoms.^17^,^18^

Because of the absence of therapies indicated for EoE, data on how patients are treated in clinical practice are limited. In addition, studies assessing health care resource use (HCRU) and quantifying the disease burden in this population are limited and outdated.^19^ The aims of this 2-phase, retrospective claims-based study were to: (1) investigate characteristics, HCRU and treatment patterns in patients with EoE in the USA before and after diagnosis; and (2) compare annualized HCRU data in a cohort of patients with EoE with that of a matched cohort of individuals without EoE.

## MATERIALS AND METHODS

### Study Design

This study analyzed US administrative claims data from the Truven Health MarketScan Commercial Claims and Encounters and Medicare Supplemental databases (now part of IBM Watson Health, Armonk, NY), from January 2008 to September 2016. The databases contain data from over 150 large employers in the USA and ∼200 different insurance companies. Since 1995, the databases have provided data for ∼240 million unique patients.^20^ Data include: enrollment history; health plan provider type (commercial only; commercial+Medicare supplement; Medicare supplement only); claims for medical (provider and institutional) and pharmacy services (National Drug Codes, days of supply and quantity dispensed); and expenditure information for insured employees and their dependents, as well as for Medicare-eligible retirees with employer-provided Medicare supplement plans. Inpatient services are summarized at the claims and stay levels.^20^ Data were anonymized and compliant with the patient requirements of the Health Insurance Portability and Accountability Act.^21^

### Study Population

#### Phase 1: Study of All Patients With EoE

The study population consisted of all patients in the database who had at least 2 diagnoses of EoE (identified using the International Classification of Diseases, 9th or 10th Revision, Clinical Modification [ICD-9/10-CM] codes 530.13 [ICD-9-CM] or K20.0 [ICD-10-CM]) documented on different dates.^22^ On the basis of the validated use of single diagnosis codes to identify patients with EoE with 95% specificity,^23^ which has been applied in a previous claims-based study,^19^ 2 diagnosis codes were used in this study, thus increasing the specificity of identification. Patients were required to have been continuously enrolled in a health plan for at least 12 months before (the preindex period) and at least 3 months after the date of their first claim associated with a diagnosis of EoE (the index date), and to have made medical and pharmacy benefit claims during both periods. Patients who switched health plans during either period were included in the analysis. Patients were followed up from the index date until the date of termination of eligibility. Patients who were enrolled in any capitated insurance plan(s) during the preindex or follow-up periods, or who had a documented diagnosis of oropharyngeal or esophageal cancer at any time before the index date or at any time during the follow-up period, were excluded from the study.

#### Phase 2: Matched Cohort Study

In the matched cohort study, the EoE population included all patients who met the inclusion criteria for phase 1 and who were continuously enrolled in a health plan for at least 12 months after the index date. The control cohort was selected from a 5% random sample of enrollees in the database. This cohort comprised individuals who had no documented diagnosis of EoE at any time in the history of the database, and who had been continuously enrolled in a health plan during the 12 months before the matched index date (the preindex period) and for 12 months after the matched index date (the study period).

Individuals in the control cohort were assigned random index dates so that the distribution of the length of time (mean and SD) between the eligibility start date and index date matched that of the EoE cohort. Individuals in the matched cohort who were enrolled in any capitated insurance plan(s) during the preindex or study periods were excluded from the study. The 2 cohorts were matched 1:1 by sex, age, geographic region (as of the index date), Charlson comorbidity index (CCI) classification during the preindex period, and length of continuous enrollment from the eligibility start date to the index date.

### Study Measures

#### Patient Demographics and Clinical Characteristics

Patient demographics (age, sex, and geographic region), index year, and health insurance plan were examined at the index date. Patient clinical characteristics [EoE-related comorbidities (identified using ICD-9/10-CM codes), CCI scores, frequency of prior drug treatments, nonpharmacotherapies, and diagnostic and allergy tests] were assessed during the preindex period.

#### Patterns of Pharmacotherapy and Use of Esophageal Dilation (Phase 1 Only)

During the follow-up period, off-label use of pharmacotherapies that were considered likely to be used to treat EoE (study drugs) was assessed at the class level [eg, PPIs, TCS (including swallowed topical fluticasone and budesonide) and other treatment classes (leukotriene antagonists such as montelukast, or monoclonal antibodies such as omalizumab, mepolizumab, and reslizumab)]. Medications classified as “other” that were prescribed before the index date were not considered treatments for EoE.

Up to 3 consecutive lines of pharmacotherapy were identified for each patient; the first off-label study drug used during the follow-up period was defined as “first-line” pharmacotherapy. First-line pharmacotherapy was assumed to commence on the date of the first claim for a study drug either on or after the index date. Second-line and third-line pharmacotherapies were defined as a patient switching to a different drug class, addition of a different drug class to an existing line of pharmacotherapy, or removal of an existing study drug. If a patient discontinued a study drug but restarted it later, the treatment was considered to occur in the same line of therapy, regardless of the gap between study drugs. A patient was considered to have received combination therapy if prescriptions for study drugs of at least 2 classes had been filled within a 30-day period; otherwise, the patient was considered to have received monotherapy.

The proportion of patients who underwent esophageal dilation during the follow-up period was also assessed.

#### HCRU

Emergency room (ER) visits (frequency and type, eg, diagnosis of dysphagia), outpatient visits (frequency and provider, eg, gastroenterologist), inpatient visits (frequency, primary diagnosis, and length of stay), and diagnostic endoscopy use were recorded during the preindex and follow-up periods. In addition, outpatient visits and diagnostic endoscopy use were recorded separately on the index date (phase 1 only).

### Statistical Analyses

Patient demographics, clinical characteristics, and HCRU were summarized using descriptive statistics [n, mean, and SD for continuous variables and n (%) for categorical variables]. Unadjusted comparisons of the matched cohorts were performed using McNemar’s test or the exact McNemar’s test for binary variables. A 2-tailed *P-*value <0.05 was considered statistically significant. Logistic regression models with generalized estimating equations, controlled for index year, health plan type at index date, comorbidities [gastroesophageal reflux disease (GERD) and atopic diseases], prior treatment (medication for GERD and asthma) and HCRU during the preindex period, were used to perform adjusted comparisons of HCRU between the matched cohorts.

## RESULTS

### All Patients With EoE

#### Preindex Demographics and Clinical Characteristics

**FIGURE 1:**
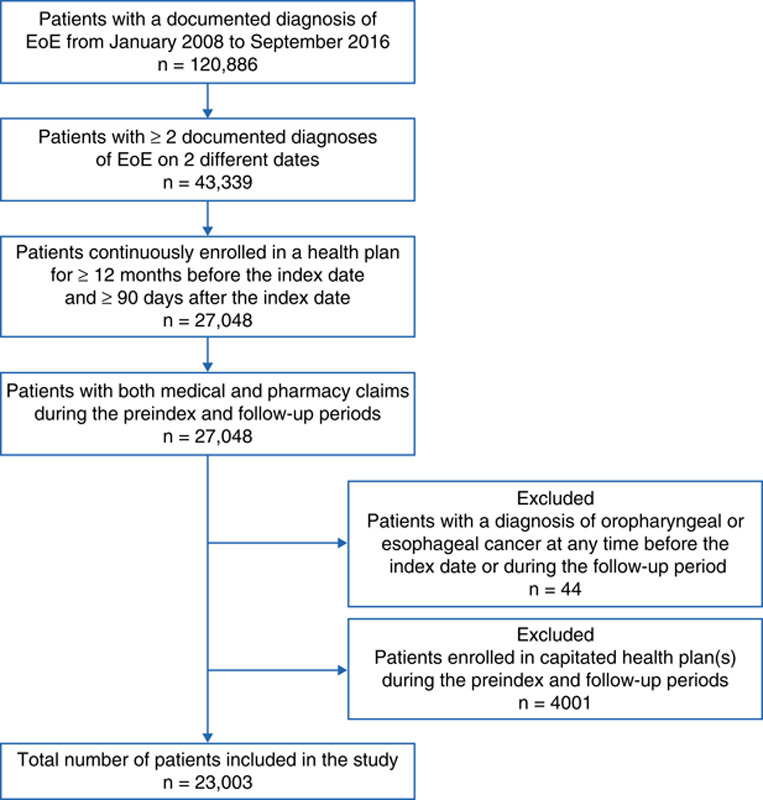
Flowchart for the selection of patients with EoE. EoE indicates eosinophilic esophagitis.

The study population comprised 23,003 patients with EoE (Fig. 1). Of these patients, 3103 (13.5%) were children (aged 0 to 11 y), 2417 (10.5%) were adolescents (aged 12 to 17 y), 14,492 (63.0%) were adults aged 18 to 54 years, and 2991 (13.0%) were adults aged ≥55 years. The mean (SD) age was 34.3 (17.9) years and the majority of patients were male (64.8%; Table 1). Additional preindex parameters (geographic region, index year, and health insurance plan) are reported in Table S1 (Supplemental Digital Content 1, http://links.lww.com/JCG/A653).

**TABLE 1:** Baseline Demographics and Clinical Characteristics of Patients With EoE*

		By Sex		By Age Group (Years)			
Characteristic	All Patients (N=23,003)	Female (n=8090)	Male (n=14,913)	0-11 (n=3103)	12-17 (n=2417)	18-54 (n=14,492)	≥55 (n=2991)
Age, years							
Mean (SD)	34.3 (17.9)	36.6 (17.7)	33.0 (17.8)	6.4 (3.2)	14.6 (1.7)	37.9 (10.0)	61.4 (6.6)
Sex, n (%)							
Female	8090 (35.2)	8090 (100)	—	871 (28.1)	719 (29.7)	5192 (35.8)	1308 (43.7)
Male	14,913 (64.8)	—	14,913 (100)	2232 (71.9)	1698 (70.3)	9300 (64.2)	1683 (56.3)
EoE-related symptom, n (%)							
Dysphagia	1786 (7.8)	625 (7.7)	1161 (7.8)	83 (2.7)	140 (5.8)	1306 (9.0)	257 (8.6)
EoE-related comorbidity, n (%)							
GERD	7963 (34.6)	3049 (37.7)	4914 (33.0)	1315 (42.4)	764 (31.6)	4767 (32.9)	1117 (37.3)
Allergic rhinitis	4479 (19.5)	1714 (21.2)	2765 (18.5)	1013 (32.6)	668 (27.6)	2377 (16.4)	421 (14.1)
Asthma	3383 (14.7)	1378 (17.0)	2005 (13.4)	845 (27.2)	538 (22.3)	1662 (11.5)	338 (11.3)
Esophageal stricture	2525 (11.0)	856 (10.6)	1669 (11.2)	41 (1.3)	113 (4.7)	1909 (13.2)	462 (15.4)
Food impaction	1789 (7.8)	495 (6.1)	1294 (8.7)	82 (2.6)	190 (7.9)	1313 (9.1)	204 (6.8)
Eczema	1662 (7.2)	662 (8.2)	1000 (6.7)	591 (19.0)	171 (7.1)	703 (4.9)	197 (6.6)
Food allergy	1001 (4.4)	336 (4.2)	665 (4.5)	552 (17.8)	200 (8.3)	224 (1.6)	25 (0.8)
CCI†							
Mean (SD)	0.33 (0.74)	0.40 (0.81)	0.29 (0.70)	0.40 (0.70)	0.30 (0.57)	0.28 (0.69)	0.55 (1.05)
Prior treatments, n (%)							
Esophageal dilation	1724 (7.5)	702 (8.7)	1022 (6.9)	16 (0.5)	31 (1.3)	1309 (9.0)	368 (12.3)
Medications for GERD	10,640 (46.3)	3990 (49.3)	6650 (44.6)	1798 (57.9)	1197 (49.5)	6254 (43.2)	1391 (46.5)
PPI	10,072 (43.8)	3774 (46.7)	6298 (42.2)	1575 (50.8)	1132 (46.8)	6034 (41.6)	1331 (44.5)
Antacids	24 (0.1)	6 (0.1)	18 (0.1)	22 (0.7)	0 (0)	2 (0.01)	0 (0)
Histamine 2-receptor antagonists	1426 (6.2)	553 (6.8)	873 (5.8)	522 (16.8)	172 (7.1)	585 (4.0)	147 (4.9)
Medications for asthma	8934 (38.8)	3495 (43.2)	5439 (36.5)	1662 (53.6)	1137 (47.0)	5114 (35.3)	1021 (34.1)
Anticholinergics	106 (0.5)	36 (0.4)	70 (0.5)	32 (1.0)	11 (0.5)	33 (0.2)	30 (1.0)
Antileukotrienes/leukotriene modifiers	1941 (8.4)	752 (9.3)	1189 (8.0)	510 (16.4)	336 (13.9)	918 (6.3)	177 (5.9)
Cromolyn sodium	54 (0.2)	23 (0.3)	31 (0.2)	17 (0.5)	16 (0.7)	19 (0.1)	2 (0.1)
Oral or topical corticosteroids	6625 (28.8)	2612 (32.3)	4013 (26.9)	1309 (42.2)	808 (33.4)	3744 (25.8)	764 (25.5)
Inhaled β2-agonists	3839 (16.7)	1540 (19.0)	2299 (15.4)	974 (31.4)	561 (23.2)	1952 (13.5)	352 (11.8)
Methylxanthines	21 (0.1)	8 (0.1)	13 (0.1)	1 (0.03)	1 (0.04)	10 (0.1)	9 (0.3)
Immunomodulators	148 (0.6)	74 (0.9)	74 (0.5)	17 (0.5)	24 (1.0)	80 (0.6)	27 (0.9)
Anticholinergic agent combinations	100 (0.4)	49 (0.6)	51 (0.3)	18 (0.6)	9 (0.4)	54 (0.4)	19 (0.6)
β2-agonist/corticosteroid combinations	1019 (4.4)	425 (5.3)	594 (4.0)	109 (3.5)	126 (5.2)	629 (4.3)	155 (5.2)
Prior diagnostic tests, n (%)							
Diagnostic endoscopy (preindex period)	6820 (29.6)	2621 (32.4)	4199 (28.2)	1106 (35.6)	775 (32.1)	3980 (27.5)	959 (32.1)
Diagnostic endoscopy (preindex period and at index)	18,918 (82.2)	6675 (82.5)	12,243 (82.1)	2580 (83.1)	2016 (83.4)	11,845 (81.7)	2477 (82.8)
Prior allergy tests, n (%)							
Allergy testing for food elimination	2715 (11.8)	989 (12.2)	1726 (11.6)	1117 (36.0)	490 (20.3)	970 (6.7)	138 (4.6)
Skin prick test	1861 (8.1)	682 (8.4)	1179 (7.9)	729 (23.5)	309 (12.8)	727 (5.0)	96 (3.2)
RAST	1356 (5.9)	479 (5.9)	877 (5.9)	664 (21.4)	276 (11.4)	363 (2.5)	53 (1.8)
Atopy patch test	173 (0.8)	57 (0.7)	116 (0.8)	79 (2.5)	35 (1.4)	51 (0.4)	8 (0.3)

* Age and sex were recorded as of the index date. All other parameters were recorded during the 12-month preindex period unless otherwise specified.

† CCI is used to predict the 1-year mortality for a patient who may have a range of comorbid conditions. Each comorbidity is assigned a score of 1, 2, 3, or 6 (higher scores indicate a greater risk of death associated with the condition), and the scores are summed to provide an overall score.^24^

CCI indicates Charlson comorbidity index; EoE, eosinophilic esophagitis; GERD, gastroesophageal reflux disease; PPI, proton pump inhibitor; RAST, radioallergosorbent test.

GERD was the most common condition recorded in the 12 months before diagnosis of EoE (34.6%), followed by allergic rhinitis (19.5%), asthma (14.7%), esophageal stricture (11.0%), food impaction (7.8%), eczema (7.2%), and food allergy (4.4%) (Table 1). Esophageal stricture was more frequently seen in adults aged ≥55 years (15.4%) and adults aged 18 to 54 years (13.2%) than in adolescents (4.7%) or children (1.3%). Atopic diseases, including allergic rhinitis, asthma, eczema, and food allergy were generally more frequently recorded in children than in adolescents or adults (Table 1). Dysphagia was reported in 7.8% of patients; the highest proportions were observed among adults aged 18 to 54 years (9.0%) and aged ≥55 years (8.6%) (Table 1). The mean (SD) CCI score was 0.33 (0.74) (Table 1). Overall, 2.1% of patients had a CCI score ≥3; the highest proportions of patients with this score were women (2.8%) and patients aged ≥55 years (5.2%).

Almost half (46.3%; n=10,640) of patients had received treatment indicated for GERD in the 12 months before diagnosis of EoE (Table 1). Of these patients, 94.7% were prescribed a PPI, 13.4% were prescribed histamine 2-receptor antagonists and 0.2% were prescribed antacids. Treatment for GERD was recorded more frequently in children (57.9%) than in adolescents (49.5%) or adult subgroups (46.5% in adults aged ≥55 years and 43.2% in adults aged 18 to 54 y).

A total of 8934 (38.8%) patients had received treatment indicated for asthma in the 12 months before diagnosis of EoE. Among these patients, the most commonly prescribed treatments for asthma were oral and inhaled corticosteroids (74.2%), inhaled β2-agonists (43.0%), antileukotrienes/leukotriene modifiers (21.7%), and inhaled β2-agonist/corticosteroid combinations (11.4%). Treatment for asthma was recorded more frequently in children (53.6%) than in adult subgroups (35.3% in adults aged 18 to 54 y and 34.1% in adults aged ≥55 years).

#### Patterns of Pharmacotherapy After Diagnosis of EoE

**FIGURE 2:**
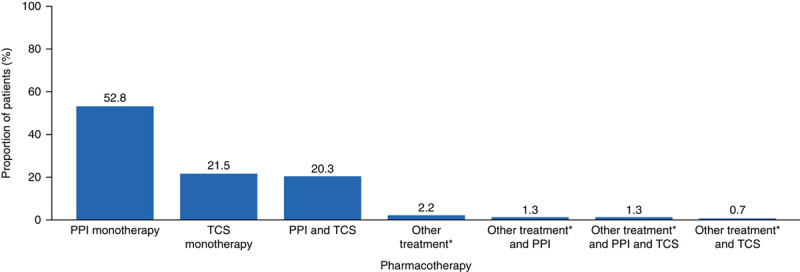
Off-label, first-line pharmacotherapy received by patients with EoE on or after diagnosis. *Other treatments included leukotriene antagonists (montelukast sodium) and monoclonal antibodies (ie, omalizumab, mepolizumab, and reslizumab). EoE indicates eosinophilic esophagitis; PPI, proton pump inhibitor; TCS, topical corticosteroids.

The mean (SD) duration of follow-up was 2.3 (1.7) years, range 0.3 to 7.8 years. Overall, 19,150 patients (83.3%) were prescribed off-label pharmacotherapy for the treatment of EoE during follow-up. PPI monotherapy was the most common off-label first-line treatment (52.8%), followed by TCS monotherapy (21.5%) and PPI and TCS combination therapy (20.3%) (Fig. 2).

**FIGURE 3:**
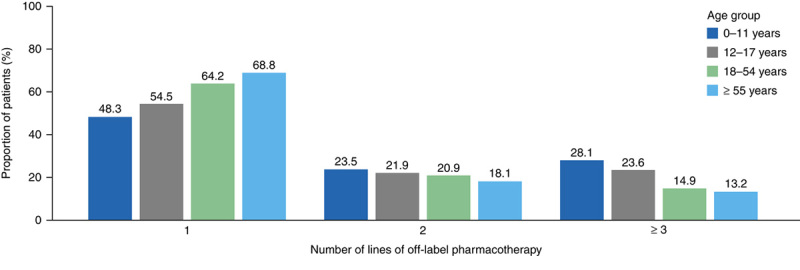
Number of lines of off-label pharmacotherapy received by patients with eosinophilic esophagitis on or after diagnosis, stratified by age group.

A total of 3336 patients (14.5%) received at least 3 lines of off-label pharmacotherapy. Children and adolescents switched study drug class more frequently than adults; 28.1% of children and 23.6% of adolescents received at least 3 lines of off-label pharmacotherapy, compared with 14.9% of adults aged 18 to 54 years and 13.2% of adults aged ≥55 years (Fig. 3).

During the follow-up period, 22.8% of patients (5246/23,003) had evidence of at least 1 esophageal dilation. This procedure was more common in adults aged 18 to 54 years (28.8%) and adults ≥55 years (28.6%) than in adolescents (7.6%) and children (0.9%).

#### HCRU

**FIGURE 4:**
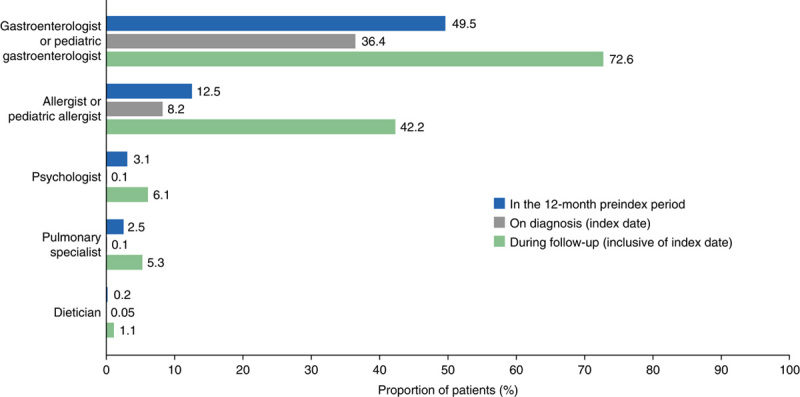
Outpatient visits in patients with eosinophilic esophagitis, by specialist.

Overall, 97.6% and 99.9% of patients with EoE had at least 1 outpatient visit before and after diagnosis, respectively. The mean (SD) number of outpatient visits among patients with EoE was 12.4 (13.5) in the 12 months before diagnosis and 14.4 (13.8) during the follow-up period. The most common outpatient visits were to gastroenterologists/pediatric gastroenterologists (preindex period, 49.5%; index date, 36.4%; follow-up period, 72.6%) and allergists/pediatric allergists (preindex period, 12.5%; index date, 8.2%; follow-up period, 42.2%) (Fig. 4).

**FIGURE 5:**
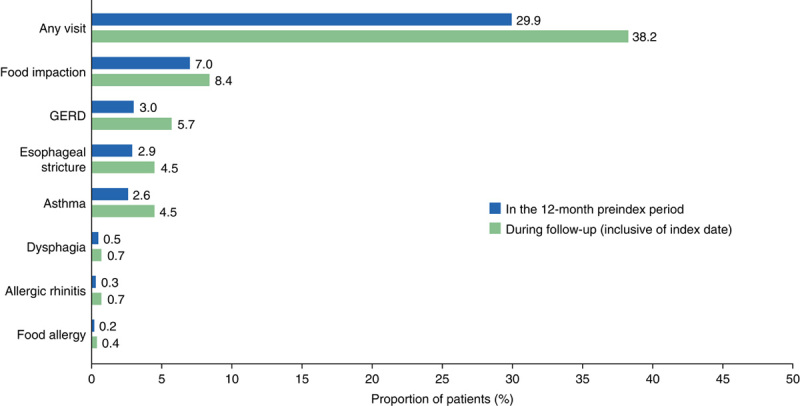
Emergency room visits in patients with eosinophilic esophagitis, stratified by associated diagnosis. GERD indicates gastroesophageal reflux disease.

Overall, 29.9% of patients (6885/23,003) had at least 1 ER visit in the 12 months before diagnosis compared with 38.2% of patients (8788/23,003) during the follow-up period. During both periods, ER visits were most commonly associated with food impaction (Fig. 5). Food impaction was more common in patients aged 18 to 54 years (preindex, 8.3%; follow-up, 10.1%) than in patients aged ≥55 years (preindex, 6.3%; follow-up, 6.9%), 12 to 17 years (preindex, 6.8%; follow-up, 8.6%), and 0 to 11 years (preindex, 2.1%; follow-up, 1.9%).

During the 12-month preindex period, 6.0% of patients (1383/23,003) had at least 1 recorded inpatient admission for any reason [mean (SD) number of inpatient days, 0.37 (2.32)] compared with 11.7% of patients (2691/23,003) during the follow-up period [mean (SD) annualized number of inpatient days, 0.52 (3.29)]. “Foreign body in the esophagus” (2.2%) and “EoE” (4.3%) were the most common diagnoses associated with inpatient admission before and after diagnosis of EoE, respectively. The mean (SD) length of stay among hospitalized patients was 6.09 (7.37) days (range: 1 to 106 d) during the preindex period compared with 4.42 (8.67) days (range: 0.13 to 172.5 d) during the follow-up period.

Overall, 29.6% of patients (6820/23,003) had claims for a diagnostic endoscopy during the preindex period (Table 1), compared with 57.6% (13,234/23,003) on the index date and 25.1% (5782/23,003) during the follow-up period (exclusive of the index date). During the follow-up period, a greater proportion of adolescents (83.8%) and children (83.6%) had claims for a diagnostic endoscopy than adults aged 18 to 54 years (76.1%) and adults aged ≥55 years (74.5%).

### Matched Cohort Study

Overall, 16,094 individuals were included in each cohort [mean (SD) age, 34.3 (17.9) years; 65.0% male] (Table S2, Supplemental Digital Content 2, http://links.lww.com/JCG/A653). The frequency and types of HCRU in each cohort are shown in Table 2. Of patients with EoE, 73.9% underwent a diagnostic endoscopy during the 12-month study period [mean (SD) number of uses, 1.01 (0.86)] compared with only 1.7% [mean (SD) number of uses, 0.02 (0.14)] in the matched cohort (*P*<0.0001). The proportion of patients with EoE who had at least 1 ER visit was approximately double that of individuals without EoE [26.2% vs. 12.9%; adjusted odds ratio (OR) 2.2; 95% confidence interval (CI), 2.0-2.3; *P*<0.0001]. In addition, all-cause inpatient admissions and outpatient visits were significantly more likely to occur in patients with EoE than in matched controls (inpatient admissions—adjusted OR: 1.5, 95% CI: 1.3-1.7, *P*<0.0001; outpatient visits—adjusted OR: 24.2, 95% CI: 18.3-32.1, *P*<0.0001).

**TABLE 2:** All-cause HCRU in Patients With EoE and Matched Controls During Follow-up

HCRU	Patients With EoE (n=16,094) [A]	Matched Controls (n=16,094) [B]	Unadjusted OR* (95% CI)	*P* †	Adjusted OR (95% CI)‡	*P* ‡ [A] vs. [B]
Diagnostic endoscopies, n (%)	11,890 (73.9)	278 (1.7)	160.9 (142.2-182.1)	<0.0001	184.3 (160.4-211.7)	<0.0001
Inpatient admissions, n (%)	1107 (6.9)	605 (3.8)	1.9 (1.7-2.1)	<0.0001	1.5 (1.3-1.7)	<0.0001
ER visits						
Any visit, n (%)	4211 (26.2)	2069 (12.9)	2.4 (2.3-2.6)	<0.0001	2.2 (2.0-2.3)	<0.0001
Food impaction	1083 (6.7)	7 (0.04)	165.8 (78.8-348.7)	<0.0001	—	<0.0001
Esophageal stricture	510 (3.2)	1 (0.006)	526.7 (74.0-3746.4)	<0.0001	—	<0.0001
Asthma	403 (2.5)	124 (0.8)	3.3 (2.7-4.1)	<0.0001	2.1 (1.7-2.7)	<0.0001
Food allergy	81 (0.5)	12 (0.1)	6.8 (3.7-12.4)	<0.0001	4.9 (2.5-9.5)	<0.0001
Dysphagia	77 (0.5)	0 (0.0)	77.4 (10.8-556.3)	<0.0001	—	<0.0001
GERD	509 (3.2)	54 (0.3)	9.7 (7.3-12.9)	<0.0001	5.8 (4.1-8.2)	<0.0001
Outpatient visits						
Any visit, n (%)	16,042 (99.7)	13,810 (85.8)	51.0 (38.7-67.2)	<0.0001	24.2 (18.3-32.1)	<0.0001
Gastroenterologist	10,838 (67.3)	723 (4.5)	43.8 (40.4-47.6)	<0.0001	35.6 (32.5-38.9)	<0.0001
Allergist	6226 (38.7)	534 (3.3)	18.4 (16.8-20.2)	<0.0001	14.9 (13.5-16.4)	<0.0001
Pulmonary specialist	511 (3.2)	291 (1.8)	1.8 (1.5-2.1)	<0.0001	1.1 (1.0-1.4)	0.1592
Psychologist	586 (3.6)	286 (1.8)	2.1 (1.8-2.4)	<0.0001	1.5 (1.3-1.8)	<0.0001

* Unadjusted ORs were evaluated for binary variables (ie, at least 1 visit). ORs for ER visits associated with dysphagia assume 1 event for matched controls.

† *P*-values were calculated using McNemar’s test or the exact McNemar’s test for binary variables. A *P*-value <0.05 was statistically significant.

‡ Adjusted ORs were evaluated for binary variables using logistic regression controlling for index year, health plan at index date, comorbidities (GERD and atopic disease), prior treatment (medication for GERD and asthma) and HCRU (inpatient, outpatient, and ER) during the preindex period. A generalized estimating equation was used to control for correlation between pairs. The adjusted models for ER visits associated with dysphagia, esophageal stricture, and food impaction did not converge owing to low numbers of events.

CI indicates confidence interval; EoE, eosinophilic esophagitis; ER, emergency room; GERD, gastroesophageal reflux disease; HCRU, health care resource use; OR, odds ratio.

## DISCUSSION

This 2-phase, retrospective, claims-based analysis provides an assessment of the real-world clinical impact of EoE in the USA. The use of a nationally representative administrative claims database enabled the examination of disease burden and real-world treatment patterns of 23,003 patients with EoE which is, to our knowledge, the largest EoE population analyzed to date.

The demographics of the overall study population (mean age: 34.3 y; 64.8% male) were representative of the reported epidemiology of EoE, which commonly affects individuals between 20 and 40 years of age and more frequently affects men than women.^25^,^26^ Before diagnosis of EoE, over one-third of patients (34.6%) had a diagnosis of GERD and 14.7% had a diagnosis of asthma. The high proportion of GERD diagnoses in the 12 months before diagnosis of EoE may be expected given the high prevalence of GERD in Western populations (20% to 40%).^27^ The prevalence of asthma (as identified by claims) in the present study was lower than that reported in a retrospective chart review of 449 patients with EoE (39.0%)^8^; however, these values may not be directly comparable because the asthma prevalence in the present study was only determined for the 12-month period before diagnosis of EoE.

In the 12 months before diagnosis of EoE, patients often experienced chronic EoE-related conditions and events that led to use of health care resources. Overall, 11% of patients had experienced esophageal stricture, which increased in frequency with increasing age, consistent with a previous retrospective study of 200 patients with EoE.^28^ In total, 29.6% of patients had undergone diagnostic endoscopy and 7.5% had upper gastrointestinal procedures for esophageal dilation before the index date. On the index date, 52.6% had undergone diagnostic endoscopy. The high frequency of diagnostic endoscopy is consistent with its recommendation as a diagnostic procedure for EoE.^16^,^29^ A low frequency of esophageal dilation may be expected before diagnosis of EoE because this procedure is not recommended as part of the diagnostic process, and is only recommended as a treatment for EoE in patients who do not respond to pharmacological therapy.^16^,^30^

This study showed that patients with EoE are primarily managed in the outpatient setting, with outpatient visits (most frequently to gastroenterologists/pediatric gastroenterologists) recorded for 99.9% of patients during the follow-up period. In contrast, inpatient visits and ER visits were only reported for 11.7% and 38.2% of patients, respectively. HCRU was higher during the follow-up period (mean 2.3 y) than the 12-month preindex period, with off-label use of pharmacotherapies recorded for 83.3% of patients. This high level of HCRU after EoE diagnosis may be owing to the limited number of treatment options currently available.

Examination of postdiagnosis treatment patterns revealed that PPIs were the most frequently prescribed off-label, first-line pharmacotherapy for EoE (53% of patients). The higher frequency of prescriptions for first-line PPI therapy relative to other regimens is aligned with recent guidelines for EoE diagnosis and management^30^ and potentially shows the recognition of PPIs as a first-line anti-inflammatory treatment option among clinicians. Alternatively, the higher frequency of prescriptions for PPI monotherapy compared with other regimens may result from some instances of PPI prescription for other conditions (eg, GERD, which was recorded in one-third of patients before EoE diagnosis); PPIs prescribed on or after the index date were defined in this analysis as treatments for EoE. The American College of Gastroenterology guidelines recommend swallowed TCS for an initial duration of 8 weeks as a first-line induction therapy for EoE;^16^ in this study, TCS monotherapy was the second most common first-line pharmacotherapy (prescribed to 21.5% of patients). It is important to note that, at present, TCS recommended for EoE often must first be compounded or mixed with a syrup to create a slurry that can be swallowed.^31^ This may result in inconsistent dosing, which could impact the effect of treatment. Many patients with EoE experienced multiple therapy switches (changes in study drug class), highlighting an unmet medical need for optimal management of this condition.

The matched cohort analysis showed that patients with EoE used significantly more health care resources than individuals without EoE. These findings are consistent with those from a systematic review of health-related quality of life and costs associated with EoE,^10^ and a previous retrospective claims analysis, which found that patients diagnosed with EoE (n=8135) consumed significantly more health care resources than sex-matched and age-matched controls (n=32,540).^19^ The present study controlled for key additional demographic and clinical variables, offering a more rigorous assessment of the impact of EoE on HCRU.

The strengths of this study lie with its population size of over 23,000 patients with EoE and the comparison of HCRU in this population with that of a healthy cohort matched for key demographic and clinical characteristics. In addition, logistic regression was used to compare the 2 cohorts and allowed for control of other confounding factors after matching. Several limitations of this study are noted. Diagnoses of EoE and comorbidities were identified using ICD-9/10-CM codes, which may be subject to under-reporting. In addition, the misclassification of drugs used to treat EoE is possible, as pharmacotherapies listed are not linked to a specific disease in the claims data. The use of pharmacotherapies may be underestimated if patients received over-the-counter treatments that are not captured in the database. Data relating to dietary therapies are not reported owing to under-reporting of this information in the databases. Outcomes from the matched cohort study may have been affected by any unobserved differences between the 2 cohorts. This study analyzed data for commercially insured patients; therefore, patient selection may have been subject to selection bias, and the findings may not be generalizable to other populations.

## CONCLUSIONS

In the USA, EoE affects individuals of all ages, and patients bear a substantial disease burden owing to chronic symptoms, comorbidities, and excess HCRU. In the absence of licensed therapies for EoE, and despite existing clinical guidelines, our findings suggest that real-world treatment patterns vary, and that many patients switch therapy frequently, highlighting the unmet need for adequate control of EoE-related symptoms in clinical practice. Treatments specifically formulated for patients with EoE may improve clinical benefits. Further research is required to determine the benefit of existing and new treatments in alleviating the clinical and economic burden of EoE.

## Supplementary Material

SUPPLEMENTARY MATERIAL

Supplemental Digital Content is available for this article. Direct URL citations appear in the printed text and are provided in the HTML and PDF versions of this article on the journal's website, www.jcge.com.
