# Supplementary material for: Disease Burden and Treatment Patterns Associated With Eosinophilic Esophagitis in the United States: A Retrospective Claims Study
Source: J Clin Gastroenterol. 2021 Jan 12;56(2):133–40. doi: 10.1097/MCG.0000000000001491 (PMC8754098; doi:10.1097/MCG.0000000000001491)
Supplement: SUPPLEMENTARY MATERIAL [file mcg-56-0133-s001.pdf]

SUPPLEMENTAL TABLE S1. Additional Pre-index Demographics of Patients With EoE

Demographic	All Patients (N = 23,003)	By Sex		By Age Group, Years			
		Female (n = 8090)	Male n = (14,913)	0–11 (n = 3103)	12–17 (n = 2417)	18–54 (n = 14,492)	≥ 55 (n = 2991)
**Geographic region, n (%)**							
Midwest	6391 (27.8)	2174 (26.9)	4217 (28.3)	819 (26.4)	678 (28.1)	4087 (28.2)	807 (27.0)
North East	4171 (18.1)	1392 (17.2)	2779 (18.6)	589 (19.0)	493 (20.4)	2570 (17.7)	519 (17.4)
South	7975 (34.7)	2905 (35.9)	5070 (34.0)	1221 (39.3)	849 (35.1)	4901 (33.8)	1004 (34.0)
West	4249 (18.5)	1543 (19.1)	2706 (18.1)	434 (14.0)	376 (15.6)	2811 (19.4)	628 (21.0)
Unknown	217 (0.9)	76 (0.9)	141 (0.9)	40 (1.3)	21 (0.9)	123 (0.8)	33 (1.1)
**Index year, n (%)**							
2009	2240 (9.7)	677 (8.4)	1563 (10.5)	546 (17.6)	322 (13.3)	1184 (8.2)	188 (6.3)
2010	2400 (10.4)	769 (9.5)	1631 (10.9)	400 (12.9)	301 (12.5)	1455 (10.0)	244 (8.2)
2011	2813 (12.2)	958 (11.8)	1855 (12.4)	392 (12.6)	319 (13.2)	1764 (12.2)	338 (11.3)
2012	3491 (15.2)	1253 (15.5)	2238 (15.0)	473 (15.2)	370 (15.3)	2214 (15.3)	434 (14.5)
2013	3469 (15.1)	1275 (15.8)	2194 (14.7)	418 (13.5)	324 (13.4)	2241 (15.5)	486 (16.3)
2014	3493 (15.2)	1247 (15.4)	2246 (15.1)	387 (12.5)	311 (12.9)	2282 (15.7)	513 (17.2)
2015	3374 (14.7)	1265 (15.6)	2109 (14.1)	316 (10.2)	324 (13.4)	2216 (15.3)	518 (17.3)
2016	1723 (7.5)	646 (8.0)	1077 (7.2)	171 (5.5)	146 (6.0)	1136 (7.8)	270 (9.0)
**Health insurance plan at index date, n (%)**							
CHDP	1978 (8.6)	688 (8.5)	1290 (8.7)	235 (7.6)	201 (8.3)	1347 (9.3)	195 (6.5)
Comprehensive	620 (2.7)	233 (2.9)	387 (2.6)	27 (0.9)	56 (2.3)	191 (1.3)	346 (11.6)
EPO	295 (1.3)	116 (1.4)	179 (1.2)	46 (1.5)	40 (1.7)	171 (1.2)	38 (1.3)
HDHP	1349 (5.9)	422 (5.2)	927 (6.2)	190 (6.1)	141 (5.8)	903 (6.2)	115 (3.8)
POS	1695 (7.4)	624 (7.7)	1071 (7.2)	263 (8.5)	178 (7.4)	1044 (7.2)	210 (7.0)
PPO	16,121 (70.1)	5704 (70.5)	10,417 (69.9)	2175 (70.1)	1680 (69.5)	10,267 (70.8)	1999 (66.8)
Unknown	945 (4.1)	303 (3.7)	642 (4.3)	167 (5.4)	121 (5.0)	569 (3.9)	88 (2.9)

CDHP indicates consumer-driven health plan; EoE, eosinophilic esophagitis; EPO, exclusive provider organization; HDHP, high-deductible health plan; POS, point-of-service; PPO, preferred provider organization.

SUPPLEMENTAL TABLE S2. Pre-index Characteristics of Patients With EoE and Matched Individuals Without EoE

Characteristic	Patients With EoE (n = 16,094)	Matched Individuals Without EoE (n = 16,094)
**Age, years**		
Mean (SD)	34.3 (17.9)	34.3 (17.9)
**Sex, n (%)**		
Female	5632 (35.0)	5632 (35.0)
Male	10,462 (65.0)	10,462 (65.0)
**Geographic region, n (%)**		
Midwest	4453 (27.7)	4453 (27.7)
North East	2958 (18.4)	2958 (18.4)
South	5662 (35.2)	5662 (35.2)
West/unknown	3021 (18.8)	3021 (18.8)
**Health insurance plan, n (%)**		
PPO	11,315 (70.3)	10,240 (63.6)
**Index year, n (%)**		
2009	1752 (10.9)	3561 (22.1)
2010	2003 (12.4)	2944 (18.3)
2011	2385 (14.8)	2373 (14.7)
2012	2478 (15.4)	2164 (13.4)
2013	2848 (17.7)	1881 (11.7)
2014	2582 (16.0)	1681 (10.4)
2015	2046 (12.7)	1490 (9.3)
**CCI score, n (%)***		
0	12,893 (80.1)	12,893 (80.1)
1	2523 (15.7)	2523 (15.7)
2	502 (3.1)	502 (3.1)
3+	176 (1.1)	176 (1.1)
**Comorbidities related to EoE, n (%)**		
GERD	5368 (33.4)	558 (3.5)
Asthma	2162 (13.4)	1657 (10.3)
Allergic rhinitis	3070 (19.1)	1217 (7.6)
Eczema	1130 (7.0)	624 (3.9)
Food allergy	725 (4.5%)	55 (0.3%)

**Prior treatments, n (%)**		
Medications for GERD	7464 (46.4)	1336 (8.3)
Medications for asthma	6259 (38.9)	3619 (22.5)
**Prior HCRU, n (%)**		
ER visits	4668 (29.0)	2252 (14.0)
Outpatient visits	15,706 (97.6)	13,821 (85.9)
Inpatient visits	910 (5.7)	678 (4.2)

*CCI is used to predict the 1-year mortality for a patient who may have a range of comorbid conditions. Each comorbidity is assigned a score of 1, 2, 3, or 6 (higher scores indicate a greater risk of death associated with the condition), and the scores are summed to provide an overall score.[31]

CCI indicates Charlson Comorbidity Index; EoE, eosinophilic esophagitis; ER, emergency room; GERD, gastroesophageal reflux disease; HCRU, healthcare resource use; PPO, preferred provider organization; SD, standard deviation.
